# Intermittent abdominal pain in IgA vasculitis

**DOI:** 10.1590/1984-0462/2022/40/2020202

**Published:** 2021-09-01

**Authors:** Izabel Mantovani Buscatti, Juliana Russo Simon, Vivianne Saraiva Leitao Viana, Tamima Mohamad Abou Arabi, Vitor Cavalcanti Trindade, Ana Carolina Cortez Maia, Lara Regina Cavalcante Melo, Bianca Pires Ihara, Nadia Emi Aikawa, Clovis Artur Silva

**Affiliations:** a Hospital das Clínicas, Faculdade de Medicina, Universidade de São Paulo, SP, Brazil.

**Keywords:** Immunoglobulin A, Abdominal pain, Henoch-Schönlein purpura, Glucocorticoid, Ranitidine, Imunoglobulina A, Dor abdominal, Púrpura Henoch-Schönlein, Glicocorticoides, Ranitidina

## Abstract

**Objective:**

To assess intermittent abdominal pain in IgA vasculitis patients and its relation to demographic data, clinical manifestations and treatments.

**Methods:**

A retrospective cohort study included 322 patients with IgA vasculitis (EULAR/PRINTO/PRES criteria) seen at the Pediatric Rheumatology Unit in the last 32 years. Sixteen patients were excluded due to incomplete data in medical charts. Intermittent abdominal pain was characterized by new abdominal pain after complete resolution in the first month of disease.

**Results:**

Intermittent abdominal pain was observed in 35/306 (11%) IgA vasculitis patients. The median time between first and second abdominal pain was 10 days (3–30 days). The main treatment of intermittent abdominal pain included glucocorticoid [n=26/35 (74%)] and/or ranitidine [n=22/35 (63%)]. Additional analysis showed that the frequency of intermittent purpura/petechiae (37 vs. 21%; p=0.027) and the median of purpura/petechiae duration [20 (3–90) vs. 14 (1–270) days; p=0.014] were significantly higher in IgA vasculitis patients with intermittent abdominal pain compared to those without. Gastrointestinal bleeding (49 vs. 13%; p<0.001), nephritis (71 vs. 45%; p=0.006), glucocorticoid (74 vs. 44%; p=0.001) and intravenous immunoglobulin use (6 vs. 0%; p=0.036) were also significantly higher in the former group. The frequency of ranitidine use was significantly higher in IgA vasculitis patients with intermittent abdominal pain *versus* without (63 vs. 28%; p<0.001), whereas the median of ranitidine duration was reduced in the former group [35 (2–90) vs. 60 (5–425) days; p=0.004].

**Conclusions:**

Intermittent abdominal pain occurred in nearly a tenth of IgA vasculitis patients, in the first 30 days of disease, and was associated with other severe clinical features. Therefore, this study suggests that these patients should be followed strictly with clinical and laboratorial assessment, particularly during the first month of disease course.

## RESUMO

**Objetivo:**

Avaliar a dor abdominal intermitente em pacientes com vasculite por IgA e sua relação com dados demográficos, manifestações clínicas e tratamentos.

**Métodos:**

Um estudo de coorte retrospectiva incluiu 322 pacientes com vasculite por IgA (critérios EULAR/PRINTO/PRES) em uma Unidade de Reumatologia Pediátrica durante 32 anos. Dezesseis pacientes foram excluídos em razão de dados incompletos. A dor abdominal intermitente foi caracterizada por nova dor abdominal difusa após resolução completa no primeiro mês da doença.

**Resultados:**

Dor abdominal intermitente foi observada em 35/306 (11%) dos pacientes com vasculite por IgA. A mediana entre a primeira e a segunda dor abdominal foi 10 dias (3–30 dias). O principal tratamento incluiu glicocorticoide [n=26/35 (74%)] e/ou ranitidina [n=22/35 (63%)]. Análises adicionais mostraram que a frequência da púrpura/petéquia intermitente (37 vs. 21%; p=0,027) e a mediana da duração da púrpura/petéquia [20 (3–90) vs. 14 (1–270) dias; p=0,014] foram significativamente maiores em pacientes com vasculite por IgA com dor abdominal intermitente em comparação com aqueles sem essa condição. Sangramento gastrointestinal (49 vs. 13%; p<0,001), nefrite (71 vs. 45%; p=0,006), uso de glicocorticoides (74 vs. 44%; p=0,001) e de imunoglobulina endovenosa (6 vs. 0%; p=0,036) também foram maiores no primeiro grupo. A frequência do uso de ranitidina foi significativamente maior em pacientes com vasculite por IgA com dor abdominal intermitente *versus* sem dor (63 vs. 28%; p<0,001), ao passo que a mediana da duração do uso de ranitidina foi reduzida no primeiro grupo [35 (2–90) vs. 60 (5–425) dias; p=0,004].

**Conclusões:**

Dor abdominal intermitente ocorreu em, aproximadamente, um décimo dos pacientes com vasculite por IgA, nos primeiros 30 dias da doença, e foi associada a manifestações clínicas graves. Este estudo sugere, portanto, que esses pacientes devem ser seguidos rigorosamente com avaliação clínica e laboratorial, principalmente durante o primeiro mês da doença.

## INTRODUCTION

Immunoglobulin A (IgA) vasculitis, previously termed as Henoch-Schönlein purpura, is the most frequent primary vasculitis reported in children and adolescents’ populations.^1^ ^–^ ^5^ This vasculitis is characterized by skin, articular, renal and gastrointestinal involvements.^1^ ^–^ ^6^

Acute onset of diffuse colicky abdominal pain has been described in up to 12% of IgA vasculitis patients at diagnosis. This gastrointestinal manifestation may be associated with serious complications, as bowel intussusception or gastrointestinal bleeding.^1^ ^,^ ^2^

Of note, recurrent or intermittent colicky abdominal pain in IgA vasculitis were seldom described in previous case reports or case series.^1^ ^,^ ^2^ ^,^ ^7^ ^–^ ^11^ However, to our knowledge, additional analysis comparing patients with *versus* patients without intermittent abdominal pain in the first month of IgA vasculitis were not carried out.

Thus, the aim of the present study was to characterize intermittent colicky abdominal pain in the first month of children and adolescents with IgA vasculitis, and to assess the possible association of demographic data, clinical manifestations, laboratorial abnormalities and treatments in patients with and without this gastrointestinal manifestation.

## METHOD

A retrospective cohort study was performed during a period of 32 years in a tertiary Pediatric Rheumatology Unit in São Paulo, Brazil. We analyzed 322 medical records. Of these, 16 were excluded due to lack of data. The remaining 306 patients fulfilled European League Against Rheumatism (EULAR), Paediatric Rheumatology International Trials Organisation (PRINTO) and Paediatric Rheumatology European Society (PRES) IgA vasculitis classification criteria.^6^ This study was approved by the Local Ethics Committee.

Demographic data, such as age at diagnosis in years, gender and body mass index (BMI), were evaluated.

Abdominal pain was defined as diffuse colicky abdominal pain with acute onset assessed by medical history and physical exam. Severe abdominal pain was described as the presence of at least one of the subsequent: abdominal angina (severe postprandial diffuse colicky abdominal pain),^12^ bowel intussusception or gastrointestinal bleeding. Intermittent abdominal pain was arbitrarily identified herein as a new episode of diffuse colicky abdominal ache after the complete resolution of the first event, both episodes occurring in the first month of disease.^10^ ^,^ ^11^ Recurrent abdominal pain was defined as another flare of abdominal pain after the first month of disease. Abdominal Doppler ultrasound was performed in those IgA vasculitis patients with gastrointestinal bleeding.^1^ The time between first and second diffuse colicky abdominal pain was also evaluated.

Intermittent purpura/petechiae were characterized as new skin lesions after total recovery of first episode, and persistent purpura/petechiae as cutaneous lesions lasting more than 30 days. The definition of arthritis was joint pain with limitation on movement or articular edema. Arthralgia was defined by articular pain without edema or limitation on motion.^1^ ^–^ ^3^ Intermittent arthralgia/arthritis, as new onset articular inflammation after total resolution.^1^ ^–^ ^3^ Orchitis was described as the presence of scrotal swelling and/or tenderness in physical exam and/or testicular Doppler ultrasound alterations.^2^ The duration of orchitis was measured in days.

Nephritis was defined as hematuria >5 red blood cells/high power field or presence of red blood cells in the urinary sediment and/or proteinuria >0.1g/m^2^/day. Nephrotic syndrome was diagnosed according to edema, serum albumin <2.5g/L and proteinuria >1g/m^2^/day.^3^ Acute kidney injury was characterized by increase in serum creatinine >2mg/dL^13^ or according to modified RIFLE (Risk, Injury, Failure, Loss of renal function and End-stage renal disease) criteria.^14^ Arterial hypertension was categorized as systolic and/or diastolic blood pressure ≥95^th^ percentile according to gender, age, and height on 3 or more occasions.^15^

Neuropsychiatric involvement was characterized by the presence of at least one of the following manifestations: headache, impaired consciousness, seizures, hemiparesis and cortical blindness.^16^ Current drugs were also recorded: prednisone/prednisolone, intravenous methylprednisolone, ranitidine, intravenous immunoglobulin (IVIG), azathioprine, cyclosporine, intravenous cyclophosphamide (IVCYC) and plasmapheresis.

Patients with IgA vasculitis were divided into two groups: with and without intermittent abdominal pain in the first month of disease course.

Results were presented as median (range) or mean±standard deviation for continuous variables, and number (%) for categorical variables. Pearson’s chi-square or Fisher’s exact tests were used to compare categorical variables. Mann-Whitney test or Student’s t-test were used to compare continuous variables. For all statistical tests, a p-value less than 0.05 was considered with statistical significance.

## RESULTS

Intermittent abdominal pain in the first month of disease was observed in 35/306 (11%) of IgA vasculitis patients, and generally reported as colicky and diffuse abdominal pain. This intermittent abdominal pain was associated with new cutaneous lesions in the first month of disease in 32/35 (91%) of IgA vasculitis patients. The median time between the first and the second episode of abdominal pain was 10 days (3–30 days). The main treatment of intermittent abdominal pain included glucocorticoid [n=26/35 (74%)] and/or ranitidine [n=22/35 (63%)]. Only one IgA vasculitis patient had recurrent abdominal pain at 5 year of disease. At that moment, he reported colicky and diffuse abdominal pain, concomitantly with new cutaneous lesions and nephritis flare.

Table 1 includes demographic data and clinical/laboratory involvements in 306 IgA vasculitis patients with intermittent abdominal pain compared to those without this condition. The frequency of intermittent purpura/petechiae (37 vs. 21%; p=0.027) and the median of purpura/petechiae duration [20 (3–90) vs. 14 (1–270) days; p=0.014] were significantly higher in IgA vasculitis patients with intermittent abdominal pain compared to those without this complication. Severe abdominal pain (60 vs. 29%; p<0.001), gastrointestinal bleeding (49 vs. 13%; p<0.001), nephritis (71 vs. 45%; p=0.006) and hematuria (56 vs. 25%; p<0.001) were significantly higher in the intermittent abdominal pain IgA vasculitis patients (Table 1).

**Table 1:** Demographic data and clinical/laboratory involvements in 306 IgA vasculitis patients with intermittent abdominal pain compared to those without this condition.

Variables at diagnosis, n (%)		With intermittent abdominal pain (n=35)	Without intermittent abdominal pain (n=271)	p-value
Demographic data				
	Age at diagnosis, years	7.3 (2.2–13.1)	6 (1.25–17.6)	0.073
	Follow-up duration, months	12 (1–180)	12 (0–180)	0.248
	Female, n=305	16/35 (46)	131/270 (48)	0.859
	Body mass index, kg/m2	15.9 (12.3–28.4)	16.1 (10.6–32.7)	0.728
Clinical/laboratorial involvements				
	Intermittent purpura/petechiae, n=303	13/35 (37)	55/268 (21)	0.027
	Purpura/petechiae duration, days	20 (3–90)	14 (1–270)	0.014
	Arthritis/arthralgia, n=305	26/35 (74)	214/270 (79)	0.499
	Intermittent arthritis/arthralgia, n=305	4/35 (4)	15/270 (6)	0.252
	Arthritis/arthralgia duration, days	4 (1–30)	5 (0–28)	0.842
	Abdominal pain, n=305	35/35 (100)	153/270 (57)	<0.001
	Severe abdominal pain, n=184	21/35 (60)	43/149 (29)	<0.001
	Gastrointestinal bleeding, n=303	17/35 (49)	34/268 (13)	<0.001
	Bowel intussusception	1/35 (3)	0/271 (0)	0.115
	Nephritis, n=296	24/34 (71)	119/262 (45)	0.006
	Arterial hypertension, n=256	7/30 (23)	32/226 (15)	0.185
	Nephrotic syndrome, n=297	1/35 (3)	3/262 (1)	0.396
	Acute kidney injury, n=291	0/34 (0)	5/257 (2)	1.000
	Leukocyturia, n=295	8/34 (23)	45/261 (17)	0.396
	Urinary casts, n=295	3/34 (8)	22/261 (8)	1.000
	Hematuria, n=295	19/34 (56)	65/261 (25)	<0.001
	Proteinuria, n=223	15/29 (52)	73/194 (38)	0.159
	Neuropsychiatric involvement, n=305	0/35 (0)	1/270 (0)	1.000
	Increased serum IgA (>255mg/dL), n=168	9/20 (45)	57/148 (38)	0.577
	Orchitis, n=305	3/35 (9)	24/270 (9)	1.000
	Orchitis duration, days	4 (3–7)	4 (1–17)	0.842

Results are presented as median (minimum value–maximum value) or n (%).

Regarding treatment, glucocorticoid (74 vs. 44%; p=0.001) and intravenous immunoglobulin use (6 vs. 0%; p=0.036) were also significantly higher in IgA vasculitis patients with *versus* without intermittent abdominal pain. The median of glucocorticoid therapy duration was higher in the first group [72.5 (1–365) vs. 40 (1–144) days; p=0.012]. The frequency of ranitidine use was significantly higher in IgA vasculitis patients with intermittent abdominal pain *versus* without (63 vs. 28%; p<0.001), whereas the median of ranitidine therapy duration was reduced in the former group [35 (2–90) vs. 60 (5–425) days; p=0.004] (Table 2).

**Table 2:** Treatment in 306 IgA vasculitis patients with intermittent abdominal pain* compared to those without this condition.

Treatments, n (%)	Number of studied patients	With intermittent abdominal pain (n=35)	Without intermittent abdominal pain (n=271)	p-value
Glucocorticoid	305	26/35 (74)	119/270 (44)	0.001
Prednisone/prednisolone (mg/kg/day)		1.8 (1–2)	1.5 (0–3)	0.801
Glucocorticoid (days of use)		72.5 (1–365)	40 (1–144)	0.012
Ranitidine	304	22/35 (63)	76/269 (28)	<0.001
Ranitidine (days of use)		35 (2–90)	60 (5–425)	0.004
Immunosuppressive agents**	305	1/35 (3)	0/270 (0)	0.115
Intravenous immunoglobulin	305	2/35 (6)	1/270 (0)	0.036

Results are presented as median (minimum value - maximum value) or n (%).

* In the first month of disease

** azathioprine, cyclosporine or intravenous cyclophosphamide.

## DISCUSSION

To our knowledge, this was the first study that demonstrated intermittent abdominal pain in the first month of disease as a rare manifestation of IgA vasculitis patients and was associated with skin manifestations, nephritis and severe gastrointestinal involvement. In addition, recurrent abdominal pain was rarely observed in IgA vasculitis patients.

The advantage of the present study was the selection of this population followed in a tertiary center in Latin America with predominance of complex patients.^17^ Furthermore, all subjects fulfilled the validated EULAR/PRINTO/PRES classification criteria^6^ and were systematically assessed according to our standardized database, as previously reported.^1^ ^,^ ^2^

At disease onset, approximately three quarters of our IgA vasculitis patients with intermittent abdominal pain had nephritis, suggesting a severe and periodic systemic involvement of this vasculitis. Indeed, nephritis is also commonly associated with IgA vasculitis flares, as reported in a study with Finland population.^18^

Additionally, intermittent abdominal pain occurred in the first 30 days of IgA vasculitis patients. Therefore, these patients should be followed strictly with clinical and laboratorial assessment, particularly during the first month of disease course.

Regarding treatment, ranitidine showed to be effective in gastrointestinal manifestations in IgA vasculitis patients, decreasing duration and severity of gastrointestinal pain and bleeding as described in a Turkish research.^19^ Our study demonstrated that, in spite of a higher use of ranitidine in IgA vasculitis patients with intermittent abdominal pain, the ranitidine use duration was reduced, suggesting that this H2-receptor antagonist seem to be a protective factor for this periodic abdominal pain.

Of note, recently US Food and Drug Administration alerts to healthcare professionals that ranitidine contains low levels of nitrosamine impurity called N-nitrosodimethylamine (NDMA).^20^ This substance is identified as a probable human carcinogen, and the risk and benefit should be individually considered for each patient.^21^ Further studies will be necessary to clarify this issue.

Moreover, glucocorticoid use is indicated for severe and complicated abdominal pain in IgA vasculitis patients.^22^ Our study demonstrated that IgA vasculitis patients with intermittent abdominal pain required this medication more frequently and for a longer period.

The median time between the first and second episode of intermittent diffuse colicky abdominal pain was short, and the vast majority of patients associated with new cutaneous lesions, indicating that this pain etiology was probably due to disease activity. The diagnosis of abdominal pain in IgA vasculitis is usually easy and rarely requires a differential diagnosis with esophagitis, gastritis or gastric ulcer.

The main limitation observed in the present study was the retrospective analysis, with potential missing data. We also did not assess the serum galactose-deficient IgA1, a well-known biomarker of IgA vasculitis associated with severe abnormalities at diagnosis.^23^ Abdominal Doppler ultrasound was only assessed in those IgA vasculitis patients with gastrointestinal bleeding.

In conclusion, intermittent abdominal pain occurred in nearly a tenth of IgA vasculitis patients, in the first 30 days of disease, and was associated with other severe clinical features. Therefore, our study suggests that these patients should be followed strictly with clinical and laboratorial assessment, particularly during the first month of disease course.
